# Idiopathic Acute Compartment Syndrome of the Leg with Incidental Deep Venous Thrombosis: A Case Report

**DOI:** 10.7759/cureus.5130

**Published:** 2019-07-12

**Authors:** Ehizogie Edigin, Hafeez Shaka

**Affiliations:** 1 Internal Medicine, John H. Stroger Jr. Hospital of Cook County, Chicago, USA

**Keywords:** acute compartment syndrome, deep vein thrombosis, fasciotomy, anticoagulation, idiopathic acute compartment syndrome, spontaneous acute compartment syndrome, acute extremity compartment syndrome

## Abstract

Acute compartment syndrome (ACS) is an emergency. The majority of cases are caused by underlying trauma, especially fractures. Idiopathic atraumatic ACS of the leg is very rare. The diagnosis and management of ACS should focus on the clinical presentation to avoid delay in fasciotomy for limb salvage. ACS of the leg can be caused by massive proximal iliofemoral thrombosis and rarely occlusive popliteal vein thrombosis with total or near total occlusion. Nonocclusive distal popliteal vein thrombosis, especially if chronic, does not cause ACS and when seen in a patient with ACS, it is likely an incidental finding rather than the cause of the compartment syndrome.

This is a case of idiopathic ACS of the right leg occurring in the presence of an incidental right chronic nonocclusive distal popliteal vein thrombosis.

## Introduction

Acute compartment syndrome (ACS) occurs when increased pressure within a compartment, bounded by unyielding fascial membranes, compromises the circulation and function of the tissues within that space [1]. This increased pressure compromises the function and with time, the viability of the limb affected. However, the diagnosis can be challenging as the symptoms associated with ACS can also be found in other often more common conditions [2]. This delay is longer in cases where there was no trauma to the limb. Fasciotomy remains the definitive treatment for ACS of the extremities irrespective of the etiology.

## Case presentation

A 24-year-old man with a remote history of polysubstance abuse presented to the emergency department with sudden onset of right lower extremity pain and swelling that started the night prior to presentation. He awoke from sleep due to severe sharp pain, 10/10 in intensity in his right lower extremity. He describes the pain as constant with waves of acute worsening as well as associated numbness and tingling. He denies chest pain, shortness of breath and palpitations. No history of trauma, prolonged immobilization, prolonged flights, animal bites, or blood clots in the past. He reports a history of snorting cocaine and heroin, most recently, one month ago. He denies any intravenous drug use.

On presentation, he had a blood pressure of 117/83 mmHg, heart rate of 97 beats per minute, respirations of 20 breaths per minute, saturating 99% on ambient air. Physical examination showed he was in significant painful distress. Examination of the right lower extremity revealed normal appearing overlying skin and leg swelling to the knee. He had an indurated calf with significant pain on palpation, which increased with dorsiflexion of the foot. He was unable to actively move the toes of his right foot. He had decreased sensation on the dorsum of his right foot. The dorsalis pedis and posterior tibial pulses were fully palpable. Labs were remarkable for WBC of 16.8, CK > 40,000, K 5.1, AST 1,161, ALT 432, LDH 1,1061 units/liter, and serum creatinine 1.5. Urinalysis showed large blood with only 2 RBCs on urine microscopy. X-ray of the right leg was negative for acute fracture and dislocation. Duplex venous ultrasound of right lower extremity showed a non-occlusive thrombus of the distal right popliteal vein.

The patient was started on IV Normal saline 200cc/hr for rhabdomyolysis, given several doses of hydromorphone and commenced on heparin drip for deep venous thrombus. The patient was subsequently transferred to the general medical floor for management of deep venous thrombus. The patient was evaluated by the medicine team about 4 hours into admission; he had persistent pain and numbness despite intravenous opioids, his right lower extremity exam showed tense compartments, decreased sensation to light touch in the dorsum and toes, and significant pain with passive movements of the right foot. The medicine team was concerned for acute compartment syndrome, and orthopedic surgery was consulted. The orthopedic service evaluated the patient and they felt the physical examination was consistent with an acute compartment syndrome. The patient was taken to the operating room for urgent right lower extremity fasciotomy.

The diagnosis of acute compartment syndrome was confirmed intraoperatively. His leukocytosis resolved and serum creatinine, CK, liver enzymes, and LDH down-trended with the treatment of rhabdomyolysis with intravenous fluids. He was taken back to the operating room on day 3 of admission for exploration, irrigation, debridement of incisions and wound vacuum change. On day 4 of admission, repeat duplex venous ultrasound revealed a chronic, non-occlusive, distal, popliteal venous thrombus. On day 5 of admission, he returned to the operating room for wound closure with a split-thickness skin graft from the right thigh and placement of Hemovac drain and wound vacuum change.

The patient's pain and symptoms improved after fasciotomy. He was cleared by physical therapy and discharged home in stable condition after a two-week hospital course.

## Discussion

The differential diagnosis of acute, unilateral leg pain is broad, and a timely diagnosis is essential, especially in limb-threatening conditions. Common considerations include deep venous thrombosis, thrombophlebitis, compartment syndrome, ruptured Baker's cyst, cellulitis, abscess, fracture and ligament rupture.

ACS is a surgical emergency and a delay in diagnosis and treatment has been associated with a higher likelihood of morbidity and mortality [3]. This is especially true in atraumatic cases, in which making a diagnosis of ACS may be more difficult [4, 5]. Common causes of atraumatic compartment syndromes have been reported including prolonged immobilization during surgery [6], insect and snake envenomation [7], hypothyroidism [8], long-standing uncontrolled diabetes [9, 10], occlusive acute deep vein thrombosis, use of anticoagulant therapy [11-13], and repetitive chronic trauma [14]. Our patient had no history of trauma and no recent history suggestive of any of the above possible etiologies leading to a delay in diagnosis.

Acute compartment syndrome as a result of venous thrombosis is an uncommon occurrence. Generally, phlegmasia cerulea dolens or massive iliofemoral proximal venous thrombosis results in elevated compartment pressures and is known to cause acute compartment syndrome. Distal popliteal vein occlusion rarely causes compartment syndrome, and when it does, it is secondary to a total or near-total occlusion of venous outflow as reported in a previously published case report [15].

Rhabdomyolysis is a rare cause of compartment syndrome. Typically, it would involve multiple extremities. Furthermore, there is usually an identifiable precipitant for rhabdomyolysis such as strenuous exercise in a patient with sickle cell trait, army fitness test, intravenous heroin injection or influenza A-induced [16-19].

The diagnosis of ACS starts with a high index of suspicion, along with identifying the salient clinical features [20]. ACS can be confirmed with invasive intra-compartmental pressure monitoring, however, this is not needed to make a diagnosis of ACS. Presence of severe pain that is not responsive to opioids and worsens with dorsiflexion, tense leg compartments and absence of skin changes or preceding trauma suggestive of a musculoskeletal injury should bring ACS into strong consideration.

The management of ACS should precede a comprehensive search for the possible etiology, especially in atraumatic cases as this delay could result in loss of limb. In this case, the finding of a nonocclusive thrombus in the right distal popliteal vein led to an incorrect diagnosis for the patient's symptoms, thus delaying the diagnosis and management of ACS.

Despite a thorough review of the clinical history, physical examination, laboratory and radiologic investigations, a definite etiology of this patient right leg acute compartment syndrome was not found. Hence, he was labeled as a case of idiopathic acute compartment syndrome.

## Conclusions

Idiopathic acute compartment syndrome is a rare entity that requires a high index of suspicion for timely diagnosis and treatment. Hallmarks of diagnosis of acute compartment syndrome include persistent and progressive pain, swelling, paresthesia, decreased sensation, tenderness, tense compartments, pain with passive movement of the joints, elevated measured compartment pressures and in late stage, paralysis and decreased or absent pulses in an extremity. A nonocclusive distal popliteal vein thrombus is not expected to cause acute compartment syndrome. Acute compartment syndrome is a surgical emergency and management includes prompt fasciotomy as a limb saving measure.
